# INCREASING DIGITAL EQUITY IN RURAL EASTERN NORTH CAROLINA

**DOI:** 10.1093/geroni/igae098.3202

**Published:** 2024-12-31

**Authors:** Abby Schwartz, Alice Richman, Leslie Cofie, Nate Mizell, Leslie Osorio

**Affiliations:** East Carolina University, Greenville, North Carolina, United States; East Carolina University, Greenville, North Carolina, United States; East Carolina University, Greenville, North Carolina, United States; East Carolina University, Greenville, North Carolina, United States; East Carolina University, Greenville, North Carolina, United States

## Abstract

Rural eastern North Carolina is largely an economically distressed region with limited access to broadband internet. In most places in the US, access to internet and technology is integral to daily life and is critical to accessing healthcare and social services, however in the 18 counties included in the present study, fewer than 25% of the households have broadband access. Residents in these counties experience a dire digital divide. We aim to bridge this divide by delivering an educational intervention and through the development of a technology (iPads, hotspots) library lending program in each of the counties. This poster presents results from the formative evaluation that was completed to identify the internet connectivity, technology, and resource needs in the study counties. Twenty-five interviews were completed with key informants including trusted leaders, heads of libraries/staff, and residents. The mean age of resident respondents was 51 years old, and most were female (70%) with all identifying as African American. Interviews were completed by telephone or virtually and lasted ~45 minutes each. Thematic analysis was used to analyze the interview transcripts, and key themes that emerged across the interviews highlighted the critical need and interest in a digital lending program, desired topics for educational trainings (e.g., online job searches, support in completing social service forms online), and ways to engage residents in the programs. The poster concludes with a discussion of how the themes informed the development of lending library and educational programming, and considerations when implementing these programs in rural settings.

